# Supplementary material for: Time-series transcriptome analysis identified differentially expressed genes in broiler chicken infected with mixed Eimeria species
Source: Front Genet. 2022 Aug 8;13:886781. doi: 10.3389/fgene.2022.886781 (PMC9393255; doi:10.3389/fgene.2022.886781)
Supplement: Supplementary file 2 [file DataSheet1.ZIP › 4dpi_KEGG.Gsea.1625100298584/KEGG_JAK_STAT_SIGNALING_PATHWAY.html]

Details for gene set KEGG\_JAK\_STAT\_SIGNALING\_PATHWAY[GSEA]

|  || Dataset | TMM\_4dpi\_gct\_format\_4dpi\_gct\_format.Class\_4dpi.cls #PC\_versus\_NC.Class\_4dpi.cls #PC\_versus\_NC\_repos |
| Phenotype | Class\_4dpi.cls#PC\_versus\_NC\_repos |
| Upregulated in class | 1 |
| GeneSet | KEGG\_JAK\_STAT\_SIGNALING\_PATHWAY |
| Enrichment Score (ES) | 0.3799381 |
| Normalized Enrichment Score (NES) | 1.4580655 |
| Nominal p-value | 0.015750563 |
| FDR q-value | 0.34752682 |
| FWER p-Value | 0.9932 |
Table: GSEA Results Summary

  

Fig 1: Enrichment plot: KEGG\_JAK\_STAT\_SIGNALING\_PATHWAY      
 Profile of the Running ES Score & Positions of GeneSet Members on the Rank Ordered List

  

| SYMBOL | TITLE | RANK IN GENE LIST | RANK METRIC SCORE | RUNNING ES | CORE ENRICHMENT || 1 | IL7 | na | 14 | 2.311 | 0.0607 | Yes |
| 2 | STAT1 | na | 78 | 1.743 | 0.1021 | Yes |
| 3 | IRF9 | na | 103 | 1.617 | 0.1434 | Yes |
| 4 | IL13RA1 | na | 255 | 1.245 | 0.1641 | Yes |
| 5 | IL10RB | na | 437 | 1.026 | 0.1763 | Yes |
| 6 | PIK3CA | na | 515 | 0.948 | 0.1953 | Yes |
| 7 | PIAS1 | na | 583 | 0.893 | 0.2136 | Yes |
| 8 | CISH | na | 611 | 0.873 | 0.2347 | Yes |
| 9 | IFNAR1 | na | 640 | 0.855 | 0.2552 | Yes |
| 10 | SOCS1 | na | 672 | 0.839 | 0.2751 | Yes |
| 11 | IL15RA | na | 677 | 0.836 | 0.2971 | Yes |
| 12 | SOCS3 | na | 686 | 0.829 | 0.3186 | Yes |
| 13 | STAT2 | na | 751 | 0.788 | 0.3344 | Yes |
| 14 | IFNGR2 | na | 965 | 0.685 | 0.3349 | Yes |
| 15 | SPRED2 | na | 1013 | 0.666 | 0.3487 | Yes |
| 16 | IFNAR2 | na | 1101 | 0.637 | 0.3585 | Yes |
| 17 | STAT3 | na | 1632 | 0.488 | 0.3271 | Yes |
| 18 | JAK2 | na | 1856 | 0.446 | 0.3203 | Yes |
| 19 | PIM1 | na | 1921 | 0.436 | 0.3267 | Yes |
| 20 | IL9R | na | 1942 | 0.433 | 0.3366 | Yes |
| 21 | GRB2 | na | 2146 | 0.397 | 0.3302 | Yes |
| 22 | IL2RB | na | 2243 | 0.382 | 0.3324 | Yes |
| 23 | IL12RB2 | na | 2301 | 0.375 | 0.3376 | Yes |
| 24 | PTPN11 | na | 2311 | 0.374 | 0.3469 | Yes |
| 25 | IL22 | na | 2388 | 0.364 | 0.3503 | Yes |
| 26 | AKT3 | na | 2430 | 0.357 | 0.3564 | Yes |
| 27 | GHR | na | 2475 | 0.350 | 0.3621 | Yes |
| 28 | TYK2 | na | 2527 | 0.344 | 0.3670 | Yes |
| 29 | SPRY3 | na | 2615 | 0.334 | 0.3686 | Yes |
| 30 | PIK3CB | na | 2649 | 0.329 | 0.3747 | Yes |
| 31 | IL15 | na | 2691 | 0.325 | 0.3799 | Yes |
| 32 | IL12RB1 | na | 2978 | 0.285 | 0.3636 | No |
| 33 | CNTFR | na | 3043 | 0.276 | 0.3656 | No |
| 34 | OSMR | na | 3269 | 0.247 | 0.3533 | No |
| 35 | STAT6 | na | 3657 | 0.202 | 0.3263 | No |
| 36 | LIFR | na | 3732 | 0.195 | 0.3253 | No |
| 37 | STAM | na | 3802 | 0.187 | 0.3245 | No |
| 38 | IL6ST | na | 3924 | 0.175 | 0.3191 | No |
| 39 | LEPR | na | 4060 | 0.162 | 0.3121 | No |
| 40 | CSF2RA | na | 4397 | 0.134 | 0.2875 | No |
| 41 | SOCS4 | na | 4632 | 0.112 | 0.2708 | No |
| 42 | STAM2 | na | 4672 | 0.109 | 0.2705 | No |
| 43 | IL22RA2 | na | 4691 | 0.107 | 0.2718 | No |
| 44 | PIAS2 | na | 4948 | 0.084 | 0.2526 | No |
| 45 | CCND1 | na | 5136 | 0.067 | 0.2387 | No |
| 46 | CBLB | na | 5257 | 0.058 | 0.2302 | No |
| 47 | AKT1 | na | 5330 | 0.052 | 0.2255 | No |
| 48 | SPRED1 | na | 5332 | 0.051 | 0.2268 | No |
| 49 | CNTF | na | 5499 | 0.037 | 0.2139 | No |
| 50 | BCL2L1 | na | 5617 | 0.024 | 0.2047 | No |
| 51 | IFNGR1 | na | 5858 | 0.005 | 0.1847 | No |
| 52 | IL7R | na | 5884 | 0.003 | 0.1827 | No |
| 53 | PIK3R2 | na | 5995 | -0.006 | 0.1736 | No |
| 54 | STAT5A | na | 6083 | -0.013 | 0.1667 | No |
| 55 | EP300 | na | 6138 | -0.018 | 0.1626 | No |
| 56 | IL10RA | na | 6148 | -0.018 | 0.1624 | No |
| 57 | SPRY2 | na | 6177 | -0.020 | 0.1606 | No |
| 58 | SOCS5 | na | 6250 | -0.027 | 0.1553 | No |
| 59 | IL11RA | na | 6383 | -0.037 | 0.1452 | No |
| 60 | PIK3R1 | na | 6590 | -0.054 | 0.1293 | No |
| 61 | IL2RA | na | 6593 | -0.054 | 0.1306 | No |
| 62 | CREBBP | na | 6837 | -0.074 | 0.1122 | No |
| 63 | JAK1 | na | 7146 | -0.103 | 0.0891 | No |
| 64 | STAT4 | na | 7227 | -0.111 | 0.0854 | No |
| 65 | SPRY1 | na | 7634 | -0.147 | 0.0553 | No |
| 66 | CRLF2 | na | 8160 | -0.194 | 0.0164 | No |
| 67 | MPL | na | 8232 | -0.202 | 0.0159 | No |
| 68 | PIAS4 | na | 8345 | -0.213 | 0.0122 | No |
| 69 | PIK3CG | na | 8485 | -0.227 | 0.0066 | No |
| 70 | IL21R | na | 8524 | -0.232 | 0.0096 | No |
| 71 | CBL | na | 8538 | -0.233 | 0.0148 | No |
| 72 | SOS2 | na | 8925 | -0.276 | -0.0102 | No |
| 73 | PIK3R5 | na | 9216 | -0.311 | -0.0262 | No |
| 74 | PTPN6 | na | 9540 | -0.354 | -0.0439 | No |
| 75 | SOCS2 | na | 9623 | -0.366 | -0.0409 | No |
| 76 | CCND2 | na | 9662 | -0.369 | -0.0342 | No |
| 77 | IL4R | na | 9699 | -0.374 | -0.0273 | No |
| 78 | IL20RA | na | 9818 | -0.389 | -0.0268 | No |
| 79 | PRLR | na | 9947 | -0.406 | -0.0266 | No |
| 80 | PIK3CD | na | 10148 | -0.437 | -0.0317 | No |
| 81 | JAK3 | na | 10459 | -0.490 | -0.0446 | No |
| 82 | CSF2RB | na | 10466 | -0.491 | -0.0319 | No |
| 83 | IL6R | na | 10961 | -0.595 | -0.0574 | No |
| 84 | IL2RG | na | 11028 | -0.617 | -0.0465 | No |
| 85 | CSF3R | na | 11170 | -0.654 | -0.0408 | No |
| 86 | CCND3 | na | 11181 | -0.657 | -0.0240 | No |
| 87 | IL13RA2 | na | 11238 | -0.677 | -0.0106 | No |
| 88 | MYC | na | 11510 | -0.814 | -0.0115 | No |
| 89 | PIK3R3 | na | 11670 | -0.936 | 0.0002 | No |
| 90 | SOS1 | na | 11780 | -1.055 | 0.0193 | No |
Table: GSEA details [plain text format]

  

Fig 2: KEGG\_JAK\_STAT\_SIGNALING\_PATHWAY      
 Blue-Pink O' Gram in the Space of the Analyzed GeneSet

  

Fig 3: KEGG\_JAK\_STAT\_SIGNALING\_PATHWAY: Random ES distribution      
 Gene set null distribution of ES for **KEGG\_JAK\_STAT\_SIGNALING\_PATHWAY**
